# (Dimethyl­phosphor­yl)methanaminium chloride

**DOI:** 10.1107/S1600536812037890

**Published:** 2012-09-08

**Authors:** Guido J. Reiss, Stefan Jörgens

**Affiliations:** a Institut für Anorganische Chemie und Strukturchemie, Lehrstuhl II: Material- und Strukturforschung, Heinrich-Heine-Universität Düsseldorf, Universitätsstrasse 1, D-40225 Düsseldorf, Germany

## Abstract

The crystal structure of the title salt, C_3_H_11_NOP^+^·Cl^−^, is primarily built from centrosymmetric dimers of two cations, connected head-to-tail by two charge-supported strong N—H⋯O hydrogen bonds, with a graph-set descriptor *R*_2_^2^(10). The chloride counter-anions connect these dimeric cationic units into chains along the *a*-axis direction.

## Related literature

For related compounds, see: Varbanov *et al.* (1987 ▶); Borisov *et al.* (1994 ▶); Kaukorat *et al.* (1997 ▶); Zagraniarsky *et al.* (2008 ▶); Kochel (2009 ▶). For a definition of the term tecton, see: Brunet *et al.* (1997 ▶); Resnati & Metrangolo (2007 ▶). For the use of anionic phosphinic acid derivatives as supra­molecular tectons, see: Glidewell *et al.* (2000 ▶); Chen *et al.* (2010 ▶). For graph-set theory and its applications, see: Etter *et al.* (1990 ▶); Bernstein *et al.* (1995 ▶); Grell *et al.* (2002 ▶). For hydrogen-bonded phosphinic acid derivatives, see: Reiss & Engel (2008 ▶); Meyer *et al.* (2010 ▶). For typical NH^+^⋯Cl^−^ hydrogen-bond parameters, see: Farrugia *et al.* (2001 ▶); Reiss & Bajorat (2008 ▶); Kovács & Varga (2006 ▶). For the DDM program used to obtain a profile fit of the powder diffraction data of a bulk sample of the title compound, see: Solovyov (2004 ▶).
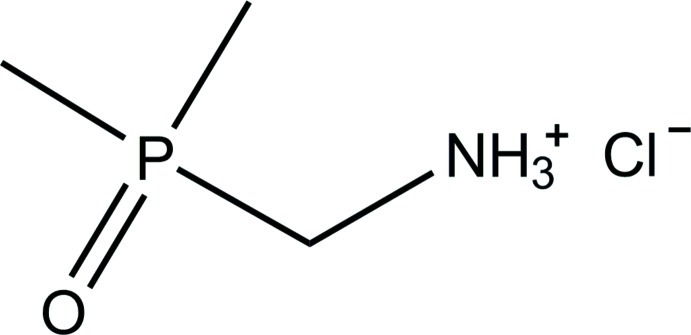


## Experimental

### 

#### Crystal data

C_3_H_11_NOP^+^·Cl^−^
*M* *_r_* = 143.55
Triclinic, 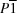

*a* = 5.2965 (2) Å
*b* = 7.7030 (4) Å
*c* = 8.8035 (3) Å
α = 84.057 (4)°
β = 87.691 (3)°
γ = 89.016 (4)°
*V* = 356.93 (3) Å^3^
*Z* = 2
Mo *K*α radiation
μ = 0.66 mm^−1^
*T* = 106 K
0.92 × 0.78 × 0.05 mm

#### Data collection

Oxford Diffraction Xcalibur diffractometer, EOS detector
Absorption correction: multi-scan (*CrysAlis PRO*; Oxford Diffraction, 2009 ▶) *T*_min_ = 0.613, *T*_max_ = 1.000
3785 measured reflections
2076 independent reflections
1968 reflections with *I* > 2σ(*I*)
*R*_int_ = 0.012

#### Refinement

*R*[*F* ^2^ > 2σ(*F* ^2^)] = 0.021
*wR*(*F* ^2^) = 0.049
*S* = 1.08
2076 reflections
93 parameters
H atoms treated by a mixture of independent and constrained refinement
Δρ_max_ = 0.48 e Å^−3^
Δρ_min_ = −0.34 e Å^−3^

### 

Data collection: *CrysAlis PRO* (Oxford Diffraction, 2009 ▶); cell refinement: *CrysAlis PRO*; data reduction: *CrysAlis PRO* program(s) used to solve structure: *SHELXS97* (Sheldrick, 2008 ▶); program(s) used to refine structure: *SHELXL97* (Sheldrick, 2008 ▶); molecular graphics: *DIAMOND* (Brandenburg, 2011 ▶); software used to prepare material for publication: *publCIF* (Westrip, 2010 ▶).

## Supplementary Material

Crystal structure: contains datablock(s) I, global. DOI: 10.1107/S1600536812037890/fj2594sup1.cif

Structure factors: contains datablock(s) I. DOI: 10.1107/S1600536812037890/fj2594Isup2.hkl

Supplementary material file. DOI: 10.1107/S1600536812037890/fj2594Isup3.cml

Additional supplementary materials:  crystallographic information; 3D view; checkCIF report

## Figures and Tables

**Table 1:** Hydrogen-bond geometry (Å, °)

*D*—H⋯*A*	*D*—H	H⋯*A*	*D*⋯*A*	*D*—H⋯*A*
N1—H1⋯Cl1	0.921 (16)	2.245 (16)	3.1367 (9)	162.8 (13)
N1—H2⋯Cl1^i^	0.872 (16)	2.262 (16)	3.1134 (9)	165.3 (14)
N1—H3⋯O1^ii^	0.905 (16)	1.791 (16)	2.6900 (12)	172.4 (15)

Symmetry codes: (i) 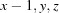; (ii) 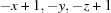.

**Fig. 1.**
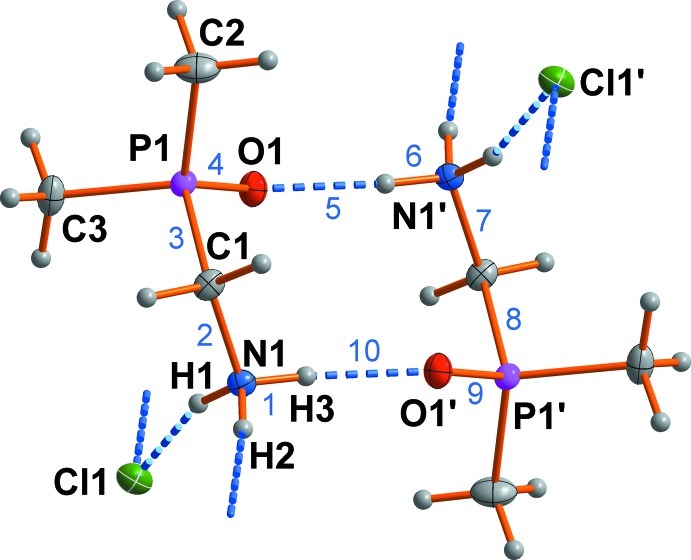
: Hydrogen-bonded dimers of the dpmaH cations connected to neighbouring chloride anions (blue numbers indicate the first-level graph-set descriptor R22(10) of the hydrogen bonded dimer; ' = 1 - x, -y, 1 - z).

**Fig. 2.**
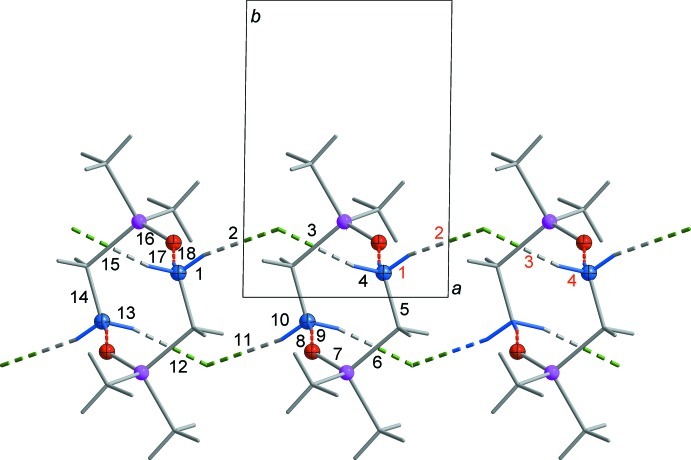
: One-dimensional chain structure of the title compound with view along [001]. Red numbers indicate the second-level graph-set C12(4) defining the connection along the backbone of the chain. Black numbers indicate the complex third-level graph-set R46(18) characterizing the ring motifs between the primary, dimeric building units.

**Fig. 3.**
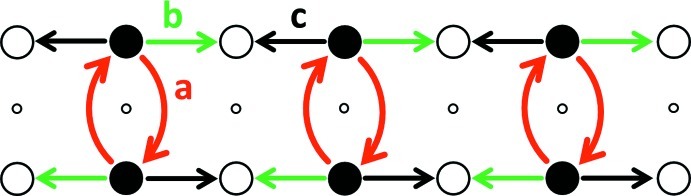
: Constructor-graph (Grell et al., 2002) of that part of the title structure shown in Fig 2. (large black dots: dpmaH cations; large open circles: chloride anions; coloured arrows a, b and c: The three crystallographically independent hydrogen bonds; small open circles: centers of inversion).

**Fig. 4.**
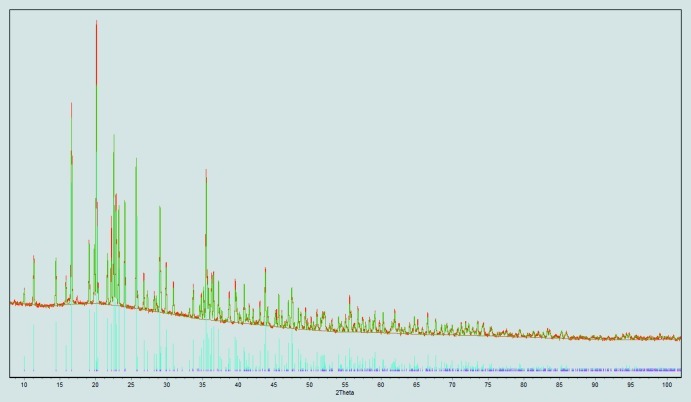
: Profile Fit (DDM programme; Solovyov, 2004) of the powder diffraction data of a bulk sample of the title compound (green line: calculated; red line: measured).

## supplementary crystallographic information

### Comment

(Dimethylphosphinyl)methanamine (*dpma*) can easily be obtained by a two-step synthesis (Varbanov *et al.*, 1987). To date only a limited number of structurally characterized *dpma* containing compounds are reported. On the one hand the structures of some transition metal complexes have been reported: Zn, Ni and Pd, (Borisov *et al.*, 1994); Cu, (Kochel, 2009). On the other hand the solid state structure of the *dpma* molecule itself has been determined (Kochel, 2009). A recent search in the *Cambridge Crystallographic Data Base* showed, that there is no structural report on the N-protonated (dimethylphosphoryl)methanaminium (*dpmaH*) so far, whereas the salt of the *N*-methylated derivative (Kaukorat *et al.*, 1997) and also more sophisticated substituated compounds are known (Zagraniarsky *et al.*, 2008). Alkyldiphosphinates have been used as *tectons* (for the term tecton, see: Brunet *et al.*, 1997; Resnati & Metrangolo, 2007) to construct hydrogen bonded frameworks (Glidewell *et al.*, 2000) and also amino phosphinic anions are known to construct hydrogen bonded one-dimensional, two-dimensional and three-dimensional supramolecular architectures (Chen *et al.*, 2010). The structure determination on the title compound is part of our continuing interest on the hydrogen bonding of methylphosphinic acids and its derivatives (Reiss & Engel, 2008) and its capability as tectons for the *crystal engineering* of new structural motifs and yet unknown species (Meyer *et al.*, 2010).

The synthesis of the title compound *dpmaHCl* succeeded by the reaction of *dpma* with concentrated hydrochloric acid. Hence, the cationic *dpmaH* features the hydrogen bond donor group NH_3_^+^ at the one end and the hydrogen bond accepting group –P=O at the other end, this *tecton* should be able to form a variety of connections among themselves and to various counter anions. In the title structure two *dpmaH* cations are connected by strong –NH^+^···O=P– hydrogen bonds (Tab. 1) head to tail to form cyclic dimers (Fig. 1; first level graph-set descriptor: *R*^2^_2_(10); Etter *et al.*, 1990; Bernstein *et al.*, 1995; Grell *et al.*, 2002). These dimers are located on centres of inversion in the triclinic space group *P*1. All bond lengths and angles in the *dpmaH* cation are in the typical range. Each dicationic cyclic dimer forms hydrogen bonds to four neighbouring chloride anions. These chloride anions form hydrogen bonds to the next dimeric unit giving an one-dimensional chain structure along [100]. The second level graph-set descriptor of this backbone-connection is C^1^_2_(4) (Fig. 2). Two *dpmaH* cations of neighbouring dimers and the two chloride anions located between them form a complex hydrogen bonded eighteen-membered ring motif (third level graph-set descriptor: *R*^4^_6_(18); Fig. 2) around a center of inversion (Fig. 2 & 3). The bond lengths of the two crystallographic independent, charge supported NH^+^···Cl^-^ hydrogen bonds are nearly identical and in the typical range for the combination of aminium groups connected to chloride anions (Farrugia *et al.*, 2001; Kovács & Varga, 2006; Reiss & Bajorat, 2008). A constructor-graph (Grell *et al.*, 2002) of exactly that part of the title structure shown in Fig. 2 is shown in Fig. 3. In this schematic diagram cations and anions are replaced by dots. Each hydrogen bond is represented by an arrow from the donor to the acceptor.

### Experimental

In a typical reaction 0.5 g (4.67 mmol) *dpma* was dissolved in 3 ml of concentrated hydrochloric acid (30–32%). The mixture was heated for a few minutes to give a clear, colourless solution. On slow cooling to room temperature colourless platelets grow from the mother liquor. The title compound is hygroscopic and storage at ambient conditions liquefies the crystalline material within a few minutes.

To check the purity of the synthesized material, powder diffraction data of a representative part of the bulk phase were collected at ambient temperature on a *Stoe Stadi P* diffractometer equipped with a PositionSensitiveDetector (flat sample, transmission, Cu *K*α1). A profile fit (Solovyov, 2004) on the powder diffraction data based on the structure model obtained from the single-crystal experiment proved the identity of the title structure with the bulk sample (Fig. 4). Only a small amount of a crystalline, unidentified impurity is present in the diffraction pattern. (T = 290 K, *a* = 5.3216 (3) Å, *b* = 7.8073 (6) Å, *c* = 8.8594 (5) Å, α = 84.591 (3) °, *β* = 87.536 (2) °, *γ* = 88.909 (3) °, *R*-DDM = 12.22; *R*-DD*M*_exp_ = 3.13; S = 3.90). – The Raman spectrum was measured using a *Bruker MULTIRAM* spectrometer (Nd:YAG-Laser at 1064 nm; RT-InGaAs-detector); 4000–70 cm^-1^: 2979(*s*), 2907 (*s*), 2844(*w*), 2631(*vw*), 2585(*vw*), 1610(*vw*), 1528(*vw*), 1449(*vw*), 1432(*m*), 1405(*w*), 1345(*vw*), 1310(*vw*, *br*), 1156(*w*), 1137(*vw*), 1091(*vw*), 1026(*w*), 946(*vw*), 920(*vw*), 895(*vw*), 859(*vw*), 786(*vw*), 725(*m*), 665(*s*), 452(*w*), 370(*w*), 321(*vw*), 285(*w*), 245(*m*), 137(*w*), 84(*m*). – IR spectroscopic data were collected on a *Digilab FT3400* spectrometer using a MIRacle ATR unit (Pike Technologies); 4000–560 cm^-1^: 3372(*m*, *br*), 2970(*s*), 2892(*s*), 2840(*s*), 2697(*m*), 2627(*m*, *sh*), 2597(*s*), 2260(*vw*), 2075(*w*), 1621(*m*), 1607(*m*), 1523(*m*), 1445(*vw*), 1422(*m*), 1407(*w*, *sh*), 1349(*vw*), 1299(*m*), 1150(*m*), 1124(*m*), 1087(*m*, *sh*), 1026(*w*), 942(*m*), 918(*m*), 889(*s*), 856(*m*), 783(*w*), 755(*w*), 723(*w*), 662(*vw*).

### Refinement

Methyl H-atoms were identified in difference syntheses, idealized and refined using rigid groups allowed to rotate about the P—C bond (AFIX 137 option of the *SHELXL97* program). The coordinates of all other H-atoms were refined freely with individual U_Iso_values.

### Crystal data

**Table:**

C_3_H_11_NOP^+^·Cl^−^	*Z* = 2
*M**_r_* = 143.55	*F*(000) = 152
Triclinic, *P*1	*D*_x_ = 1.336 Mg m^−^^3^
Hall symbol: -P 1	Mo *K*α radiation, λ = 0.71073 Å
*a* = 5.2965 (2) Å	Cell parameters from 3569 reflections
*b* = 7.7030 (4) Å	θ = 3.3–32.6°
*c* = 8.8035 (3) Å	µ = 0.66 mm^−^^1^
α = 84.057 (4)°	*T* = 106 K
β = 87.691 (3)°	Plate, colourless
γ = 89.016 (4)°	0.92 × 0.78 × 0.05 mm
*V* = 356.93 (3) Å^3^	

### Data collection

**Table:**

Oxford Diffraction Xcalibur diffractometer, Eos	2076 independent reflections
Radiation source: fine-focus sealed tube	1968 reflections with *I* > 2σ(*I*)
Graphite monochromator	*R*_int_ = 0.012
Detector resolution: 16.2711 pixels mm^-1^	θ_max_ = 30.0°, θ_min_ = 3.4°
ω scans	*h* = −4→7
Absorption correction: multi-scan (*CrysAlis PRO*; Oxford Diffraction, 2009)	*k* = −10→10
*T*_min_ = 0.613, *T*_max_ = 1.000	*l* = −12→12
3785 measured reflections	

### Refinement

**Table:**

Refinement on *F*^2^	Secondary atom site location: difference Fourier map
Least-squares matrix: full	Hydrogen site location: difference Fourier map
*R*[*F*^2^ > 2σ(*F*^2^)] = 0.021	H atoms treated by a mixture of independent and constrained refinement
*wR*(*F*^2^) = 0.049	*w* = 1/[σ^2^(*F*_o_^2^) + (0.012*P*)^2^ + 0.2*P*] where *P* = (*F*_o_^2^ + 2*F*_c_^2^)/3
*S* = 1.08	(Δ/σ)_max_ < 0.001
2076 reflections	Δρ_max_ = 0.48 e Å^−^^3^
93 parameters	Δρ_min_ = −0.34 e Å^−^^3^
0 restraints	Extinction correction: *SHELXL97* (Sheldrick, 2008), Fc^*^=kFc[1+0.001xFc^2^λ^3^/sin(2θ)]^-1/4^
Primary atom site location: structure-invariant direct methods	Extinction coefficient: 0.012 (2)

### Special details

**Table:**

Experimental. Absorption correction: CrysAlisPro, Agilent Technologies, Version 1.171.35.21 Empirical absorption correction using spherical harmonics, implemented in SCALE3 ABSPACK scaling algorithm.
Geometry. All e.s.d.'s (except the e.s.d. in the dihedral angle between two l.s. planes) are estimated using the full covariance matrix. The cell e.s.d.'s are taken into account individually in the estimation of e.s.d.'s in distances, angles and torsion angles; correlations between e.s.d.'s in cell parameters are only used when they are defined by crystal symmetry. An approximate (isotropic) treatment of cell e.s.d.'s is used for estimating e.s.d.'s involving l.s. planes.
Refinement. Refinement of *F*^2^ against ALL reflections. The weighted *R*-factor *wR* and goodness of fit *S* are based on *F*^2^, conventional *R*-factors *R* are based on *F*, with *F* set to zero for negative *F*^2^. The threshold expression of *F*^2^ > σ(*F*^2^) is used only for calculating *R*-factors(gt) *etc*. and is not relevant to the choice of reflections for refinement. *R*-factors based on *F*^2^ are statistically about twice as large as those based on *F*, and *R*- factors based on ALL data will be even larger.

### Fractional atomic coordinates and isotropic or equivalent isotropic displacement parameters (Å^2^)

**Table:**

	*x*	*y*	*z*	*U*_iso_*/*U*_eq_	
Cl1	0.83052 (4)	−0.22956 (3)	0.16135 (3)	0.01445 (7)	
P1	0.48926 (5)	0.25417 (3)	0.28914 (3)	0.00963 (7)	
O1	0.65884 (14)	0.18353 (10)	0.41347 (8)	0.01364 (15)	
N1	0.31590 (16)	−0.08170 (11)	0.28582 (10)	0.01068 (16)	
H1	0.465 (3)	−0.105 (2)	0.2342 (17)	0.024 (4)*	
H2	0.192 (3)	−0.142 (2)	0.2566 (18)	0.023 (4)*	
H3	0.338 (3)	−0.112 (2)	0.3865 (19)	0.025 (4)*	
C1	0.23746 (18)	0.10462 (13)	0.26016 (12)	0.01177 (18)	
H1A	0.099 (3)	0.1224 (18)	0.3275 (16)	0.017 (3)*	
H1B	0.185 (3)	0.128 (2)	0.1563 (18)	0.022 (4)*	
C2	0.3235 (2)	0.45000 (15)	0.32696 (15)	0.0207 (2)	
H2A	0.4425	0.5416	0.3322	0.036 (5)*	
H2B	0.2293	0.4306	0.4225	0.031 (4)*	
H2C	0.2098	0.4830	0.2465	0.029 (4)*	
C3	0.6544 (2)	0.29523 (15)	0.10879 (12)	0.0159 (2)	
H3A	0.7423	0.1910	0.0845	0.028 (4)*	
H3B	0.7737	0.3868	0.1135	0.030 (4)*	
H3C	0.5361	0.3300	0.0312	0.020 (4)*	

### Atomic displacement parameters (Å^2^)

**Table:**

	*U*^11^	*U*^22^	*U*^33^	*U*^12^	*U*^13^	*U*^23^
Cl1	0.00962 (11)	0.01729 (12)	0.01731 (13)	−0.00050 (8)	−0.00147 (8)	−0.00548 (9)
P1	0.00893 (12)	0.00947 (12)	0.01030 (12)	0.00069 (8)	−0.00136 (8)	0.00023 (8)
O1	0.0127 (3)	0.0166 (4)	0.0112 (3)	0.0006 (3)	−0.0029 (3)	0.0011 (3)
N1	0.0099 (4)	0.0110 (4)	0.0113 (4)	−0.0007 (3)	−0.0019 (3)	−0.0013 (3)
C1	0.0085 (4)	0.0118 (4)	0.0146 (5)	0.0006 (3)	−0.0016 (3)	0.0008 (3)
C2	0.0192 (5)	0.0141 (5)	0.0296 (6)	0.0047 (4)	−0.0037 (4)	−0.0056 (4)
C3	0.0131 (5)	0.0222 (5)	0.0117 (5)	−0.0035 (4)	−0.0010 (4)	0.0025 (4)

### Geometric parameters (Å, º)

**Table:**

P1—O1	1.4966 (7)	C1—H1A	0.941 (15)
P1—C3	1.7832 (11)	C1—H1B	0.965 (15)
P1—C2	1.7866 (11)	C2—H2A	0.9600
P1—C1	1.8199 (10)	C2—H2B	0.9600
N1—C1	1.4844 (13)	C2—H2C	0.9600
N1—H1	0.921 (16)	C3—H3A	0.9600
N1—H2	0.872 (16)	C3—H3B	0.9600
N1—H3	0.905 (16)	C3—H3C	0.9600
			
O1—P1—C3	112.50 (5)	N1—C1—H1B	108.5 (9)
O1—P1—C2	114.00 (5)	P1—C1—H1B	108.3 (9)
C3—P1—C2	107.74 (6)	H1A—C1—H1B	109.1 (13)
O1—P1—C1	112.45 (4)	P1—C2—H2A	109.5
C3—P1—C1	105.97 (5)	P1—C2—H2B	109.5
C2—P1—C1	103.48 (5)	H2A—C2—H2B	109.5
C1—N1—H1	112.4 (9)	P1—C2—H2C	109.5
C1—N1—H2	106.4 (10)	H2A—C2—H2C	109.5
H1—N1—H2	111.5 (14)	H2B—C2—H2C	109.5
C1—N1—H3	109.8 (10)	P1—C3—H3A	109.5
H1—N1—H3	107.6 (13)	P1—C3—H3B	109.5
H2—N1—H3	109.1 (14)	H3A—C3—H3B	109.5
N1—C1—P1	113.12 (7)	P1—C3—H3C	109.5
N1—C1—H1A	107.9 (9)	H3A—C3—H3C	109.5
P1—C1—H1A	109.8 (9)	H3B—C3—H3C	109.5
			
O1—P1—C1—N1	−34.32 (9)	C2—P1—C1—N1	−157.80 (8)
C3—P1—C1—N1	88.97 (8)		

### Hydrogen-bond geometry (Å, º)

**Table:**

*D*—H···*A*	*D*—H	H···*A*	*D*···*A*	*D*—H···*A*
N1—H1···Cl1	0.921 (16)	2.245 (16)	3.1367 (9)	162.8 (13)
N1—H2···Cl1^i^	0.872 (16)	2.262 (16)	3.1134 (9)	165.3 (14)
N1—H3···O1^ii^	0.905 (16)	1.791 (16)	2.6900 (12)	172.4 (15)

Symmetry codes: (i) *x*−1, *y*, *z*; (ii) −*x*+1, −*y*, −*z*+1.
